# Expression of UV-Sensitive Parapinopsin in the Iguana Parietal Eyes and Its Implication in UV-Sensitivity in Vertebrate Pineal-Related Organs

**DOI:** 10.1371/journal.pone.0039003

**Published:** 2012-06-14

**Authors:** Seiji Wada, Emi Kawano-Yamashita, Mitsumasa Koyanagi, Akihisa Terakita

**Affiliations:** Department of Biology and Geosciences, Graduate School of Sciences, Osaka City University, Osaka, Japan; University of Sussex, United Kingdom

## Abstract

The pineal-related organs of lower vertebrates have the ability to discriminate different wavelengths of light. This wavelength discrimination is achieved through antagonistic light responses to UV or blue and visible light. Previously, we demonstrated that parapinopsin underlies the UV reception in the lamprey pineal organ and identified parapinopsin genes in teleosts and frogs of which the pineal-related organs were reported to discriminate light. In this study, we report the first identification of parapinopsin in the reptile lineage and show its expression in the parietal eye of the green iguana. Spectroscopic analysis revealed that iguana parapinopsin is a UV-sensitive pigment, similar to lamprey parapinopsin. Interestingly, immunohistochemical analyses using antibodies specific to parapinopsin and parietopsin, a parietal eye green-sensitive pigment, revealed that parapinopsin and parietopsin are colocalized in the outer segments of the parietal eye photoreceptor cells in iguanas. These results strongly suggest that parapinopsin underlies the wavelength discrimination involving UV reception in the iguana parietal eye. The current findings support the idea that parapinopsin is a common photopigment underlying the UV-sensitivity in wavelength discrimination of the pineal-related organs found from lampreys to reptiles.

## Introduction

Lower vertebrates can discriminate between different wavelengths of light in their pineal and pineal-related organs, independent of image-forming color vision in the eyes. This wavelength discrimination is achieved through chromatic antagonism; i.e., opposing responses elicited by UV/blue and longer-wavelength light [1], [2]. In the lamprey [3] and teleost [4], [5] pineal organs, as well as the frog frontal organ [6], such wavelength discrimination through rationing between UV and visible light has been observed. The same applies to the reptilian parietal eye, which detects the ratio of UV/blue to longer-wavelength light [7]–[10].

We have recently found that parapinopsin, first identified in the catfish pineal and parapineal organs [11], is indeed the UV-sensitive photopigment in the pineal of the lamprey, one of the most primitive vertebrates [12]. We found the same for trout and clawed frog [12]. Thus, parapinopsin may be a common denominator for UV-sensing in the wavelength discrimination of the pineal-related organs of lower vertebrates. It is of interest to know whether the same is true for reptiles.

Unlike the lamprey and teleost pineal organ, where wavelength discrimination is thought to be achieved by ganglion cells involving synaptic inputs from multiple types of photoreceptor cells each having different wavelength sensitivity [1]–[6], in the reptilian parietal eye, as in the side-blotched lizard (*Uta stansburiana*), chromatic antagonism resides in the photoreceptors themselves, involving wavelength-specific hyperpolarizing and depolarizing responses to light [10]. In the latter case, the wavelength discrimination was effected by the blue-sensitive pinopsin and the green-sensitive parietopsin co-present in a single photoreceptor cell and generating antagonistic light responses [13]. Because a previous ERG study indicated that the parietal eye of green iguana (*Iguana iguana*) and the green anole (*Anolis carolinensis*) can likewise sense UV light [7], we asked whether parapinopsin is also involved in this function. This issue is also interesting from a viewpoint of evolutionary linkage between parapineal organs and parietal eyes because it was proposed that the lizard parietal eye is homologous to the parapineal organ containing parapinopsin in other lower vertebrates [14], [15].

## Materials and Methods

### Ethics Statement

This experiment was approved by the Osaka City University animal experiment committee (#S0005) and complied with the Regulations on Animal Experiments from Osaka City University.

### Animals

Green iguanas (*Iguana iguana*) were commercially obtained. The iguanas were maintained at 25°C with a 12D/12L light cycle prior to the experiments.

**Figure 1:**
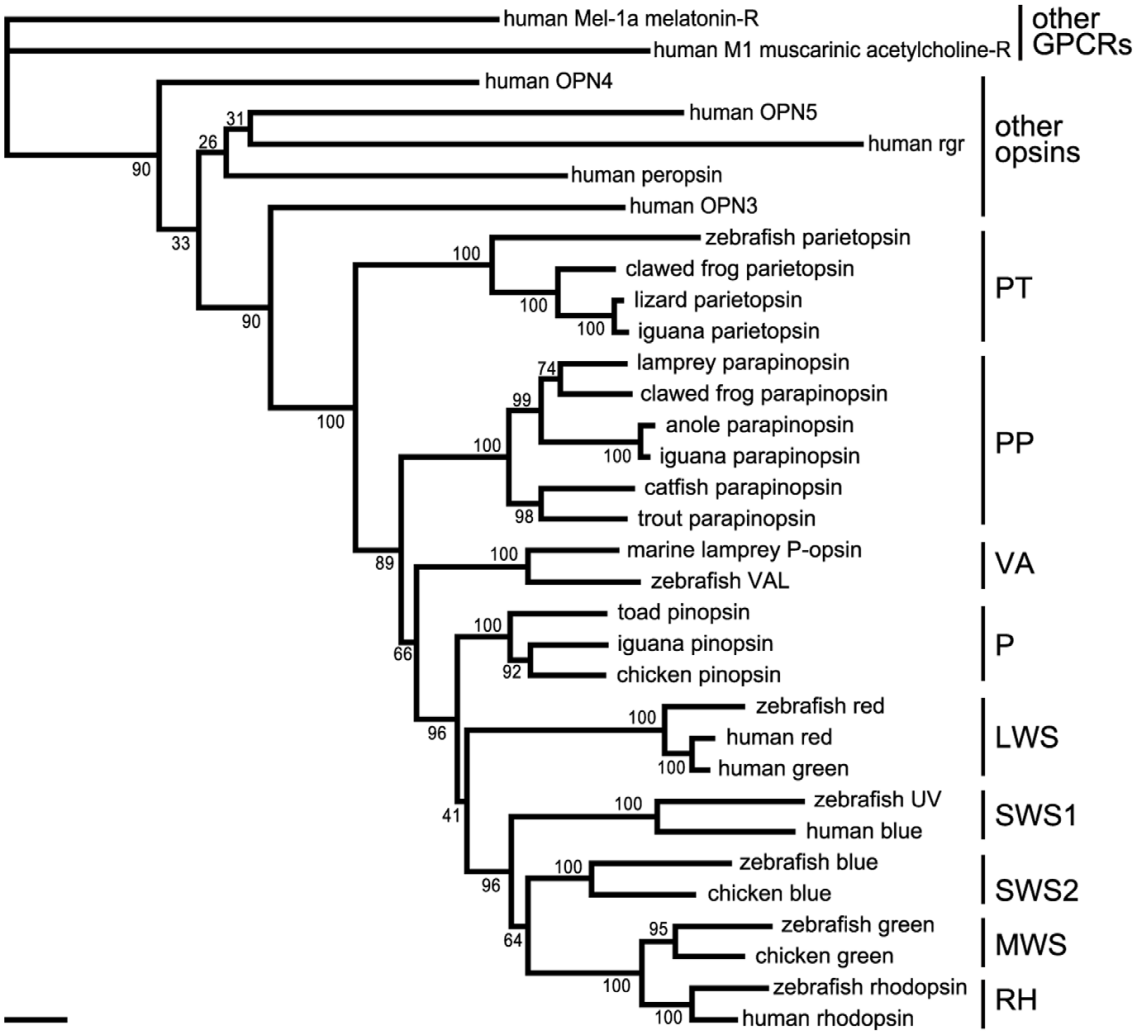
Phylogenetic positions of iguana opsins. The tree was inferred by the neighbor-joining method based on a comparison of the alignment of conserved regions, using other GPCRs as an outgroup. The bootstrap probabilities are indicated at each branch node. The names of evolutionarily and functionally classified groups are shown to the right of each cluster. The scale bar indicates 0.1 substitutions per site. Accession numbers of the sequence data from the DDBJ/EMBL/GenBank database are as follows: human Mel-1a melatonin-R (receptor), U14108; human M1 muscarinic acetylcholine-R (receptor), Y00508; human OPN4, AF147788; human OPN5, BC126198; human rgr, U15790; human peropsin, AF012270; human OPN3, AF140242; zebrafish parietopsin, XM_003201434; clawed frog parietopsin, NM_001045791; lizard parietopsin, DQ100320; iguana parietopsin, AB626970; lamprey parapinopsin, AB116380; clawed frog parapinopsin, AB159672; anole parapinopsin, AB626968; iguana parapinopsin, AB626969; catfish parapinopsin, AF028014; trout parapinopsin, AB159673; marine lamprey P-opsin, U90671; zebrafish VAL, AB035277; toad pinopsin, AF200433; iguana pinopsin, AB626971; chicken pinopsin, U15762; zebrafish red, NM_131175; human red, AH005298; human green, AH005296; zebrafish UV, NM_131319; human blue, AH003620; zebrafish blue, NM_131192; chicken blue, M92037; zebrafish green, NP_571329; chicken green, M88178; zebrafish rhodopsin, NM_131084; human rhodopsin, U49742.

### Isolation and Sequencing of Opsin cDNAs

The total RNAs extracted from the parietal eye, pineal organ, retina and brain of iguana were reverse transcribed to cDNAs using oligo (dT) primers. These cDNAs were used as the templates for PCR amplification. The sense and antisense degenerate primers that were used for obtaining the cDNA fragments of opsins from the iguana were as follows: the sense primer 5′-CCICCI(C/T)TNTT(C/T)GGNTGGGG-3′, corresponding to the amino acid sequence PP(F/L)FGWG, and the antisense primer 5′-GTNGC(A/G)TAIGGNA(A/G)CCA(A/G)CA-3′, corresponding to CW(L/F)PYAT, were used to obtain the cDNA fragment of iguana parapinopsin; the sense primer 5′-TA(C/T)GGICCIGA(A/G)GGNGTNCA-3′, corresponding to the amino acid sequence YGPEGV(H/Q), and the antisense primer 5′-ATDATIGGRTTRTANACIGG-3′, corresponding to PVYNPI(I/M), were used to obtain the cDNA fragment of iguana parietopsin; the sense primer 5′-GCGGTACITIGTIRTNTGYARRCC-3′, corresponding to the amino acid sequence RY(F/L/I/M/V)V(I/M/V)C(K/R)P, and the antisense primer 5′-CTGAATTCCAICCIAINANIGGNGG-3′, corresponding to PP(Y/H/Q/N/D/E) (Y/H/Q/N/D/E)GWNS, were used to obtain the cDNA fragment of iguana pinopsin and the sense primer 5′-GAYTGGTAYACNGTIGGNAC-3′, corresponding to the amino acid sequence DWYTVGT, and the antisense primer 5′-CGRTTRTTNACCATRTACAT-3′, corresponding to MYMVNNR, were used to obtain the cDNA fragment of iguana UV cone pigment. These primers were designed based on the genome sequences of the green anole (*Anolis carolinensis*) Genome Project at the Broad Institute (www.broadinstitute.org). The PCR amplifications were performed at an annealing temperature of 42 or 46°C. The full-length cDNAs of the opsins, that is, parapinopsin, parietopsin and pinopsin, were obtained by 3′ RACE and 5′ RACE systems (Invitrogen) (Figure S1). The nucleotide sequence of the anole parapinopsin was confirmed by cDNA cloning.

**Figure 2:**
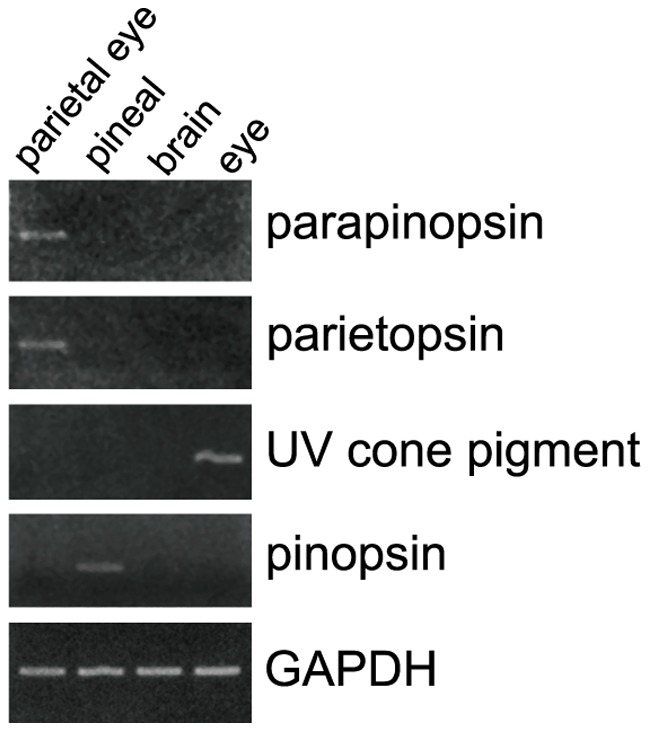
Opsin expression in various tissues of green iguana. RT-PCR analyses indicate that the iguana parapinopsin gene is expressed in the parietal eye, and the iguana UV cone pigment gene is detected only in the eye. The iguana parietopsin gene is expressed in the parietal eye. The iguana pinopsin gene is detected in the pineal organ but not in the parietal eye. The GAPDH gene was used as an internal standard.

### Phylogenetic Tree Inference

Multiple alignment of the amino acid sequences of opsins was conducted with the aid of the XCED software [16]. The molecular phylogenetic tree was inferred by the neighbor-joining method [17]. Bootstrap analysis was conducted by the method of Felsenstein [18].

**Figure 3:**
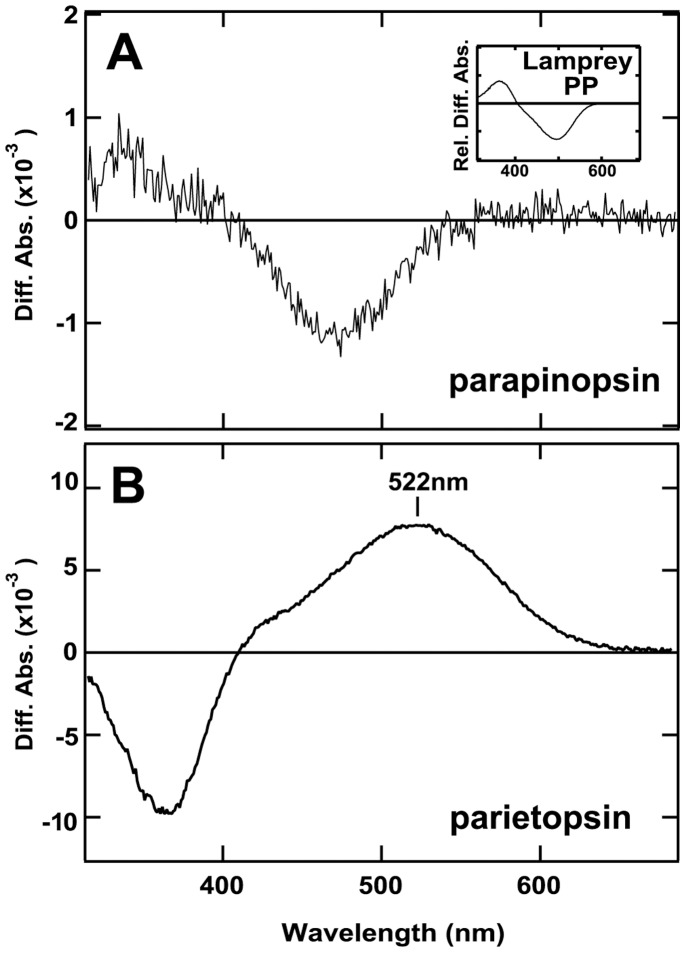
The difference absorption spectra of iguana parapinopsin and parietopsin. (A) The detergent-extract of the HEK 293 cells expressing parapinopsin was irradiated with UV light at pH 6.5. The difference spectrum of before minus after the irradiation shows that an absorption maximum of the non-irradiated parapinopsin is in the UV region. (Inset) The relative difference absorption spectrum of lamprey parapinopsin. (B) The detergent extract of iguana parietopsin-expressing cells was irradiated with green light in the presence of 50 mM hydroxylamine at pH 6.5. The difference spectrum of before minus after irradiation shows that an absorption maximum of non-irradiated parietopsin is at 522 nm.

### RT-PCR Analyses

To obtain an internal standard for normalizing the expression levels of the iguana parapinopsin (AB626969; all of the accession numbers are for DDBJ/EMBL/GenBank), parietopsin (AB626970), pinopsin (AB626971) and UV cone pigment (AB626972), we cloned glyceraldehyde 3-phosphate dehydrogenase (GAPDH, AB691541), a housekeeping gene, from iguana with the following degenerate primers: the sense primer, 5′-CCIISIGCIGAYGCNCCNATGTT-3′, and the antisense primer, 5′-GTAICCRHAYTCRTTRTCRTACCA-3′. Gene-specific PCR amplifications were performed with the following primer pairs: 5′-TGATTGGAGTCGCTATCTCC-3′ and 5′-GTTGAACCTCGCTGTGTTAC-3′ for iguana parapinopsin; 5′-CCTGTTCTATTGGCTGGGAA-3′ and 5′-ATGTTGGGATAGTTGCAGCC-3′ for iguana parietopsin; 5′-GGGCCCTTTGAAGGGCCACA-3′ and 5′-TCAGTGCGTGCCTTTGCTGG-3′ for iguana pinopsin; 5′-CAAGTACAAGAGCGAGTACT-3′ and 5′-GTAAATGATGGGGTTGTAGA-3′ for iguana UV cone pigment; and 5′-GCATTGTGGAAGCCCTTATG-3′ and 5′-GCAATGCCAGCAGCTGCATC-3′ for iguana GAPDH. The optimal annealing temperature was 60°C for all primer pairs.

**Figure 4:**
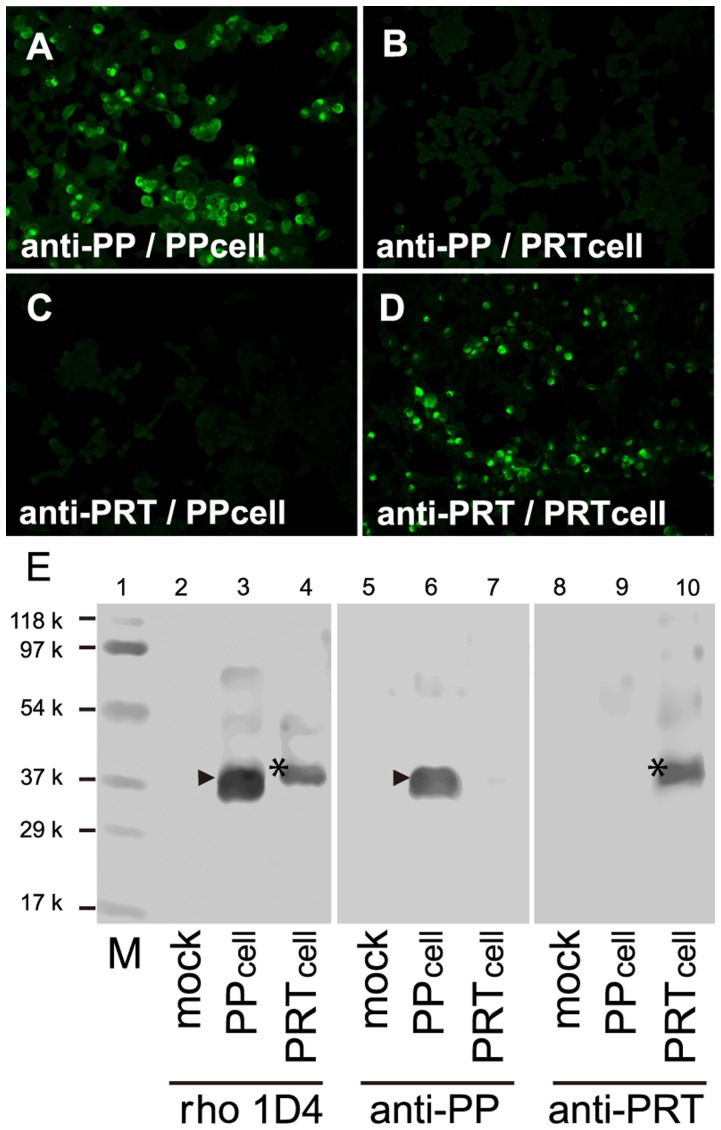
The specificity of the antibodies to parapinopsin and parietopsin. The anti-iguana parapinopsin antibody (anti-PP) labels the cells expressing iguana parapinopsin (A, PP cells) but not those expressing iguana parietopsin (B, PRT cells). The anti-iguana parietopsin antibody (anti-PRT) labels PRT cells (C) but not PP cells (D). (E) Immunoblot profiles showing that anti-PP and anti-PRT antibodies specifically recognize parapinopsin and parietopsin expressed in cultured cells. Monoclonal antibody rho 1D4 stains iguana parapinopsin (arrowhead) and parietopsin (asterisk) tagged with rho 1D4 epitope, in SDS-extracts form PP and PRT cells (lanes 3 and 4) but not from mock transfected cells (lane 2). Anti-PP specifically stained ∼38 k peptide (lane 6, arrowhead), which is the same as the band stained by rho 1D4 (lane 3), in PP cells but not in mock cells (lane 5) or PRT cells (lane 7). Anti-PRT antibodies recognize specifically ∼39 k peptide (lane 10, asterisk), which is identical to the stained band by rho 1D4 (lane 4) in PRT cells, but not in mock cells (lane 8) or PP cells (lane 9). M indicates Molecular weight standard markers (lane 1) (Bio-Rad Laboratories).

### Expression of Opsin-based Pigment

The cDNAs of iguana parapinopsin and parietopsin were tagged with the epitope sequence for the monoclonal antibody rho 1D4 (ETSQVAPA). The tagged cDNA was inserted into the plasmid vector pcDNA3.1 (Invitrogen). The pigment expression in HEK293S cells was performed as described previously [12]. Briefly, to reconstitute the pigment, the expressed proteins were incubated with excess 11-*cis* retinal overnight. The pigments were then extracted with a detergent, 1% dodecyl β-D-maltoside, in 50 mM HEPES buffer (pH 6.5). The absorption spectra of iguana parapinopsin and parietopsin were recorded at 0°C with a Shimadzu UV-2450 spectrophotometer. The detergent-extract containing parapinopsin was irradiated with UV light, which was supplied by a 1-kW halogen lamp (Philips) with a UV glass filter transmitting from ∼300 nm to ∼400 nm light with maximum transmission at 360 nm, UTVAF-50S-36U (Sigma Koki Co., Ltd.). The detergent extract-containing parietopsin was irradiated with orange light, which was supplied by a 1-kW halogen lamp (Philips) with an O-53 cutoff filter transmitting above ∼540 nm (Toshiba) in the presence of 50 mM hydroxylamine to bleach the photoproduct at pH 6.5.

**Figure 5:**
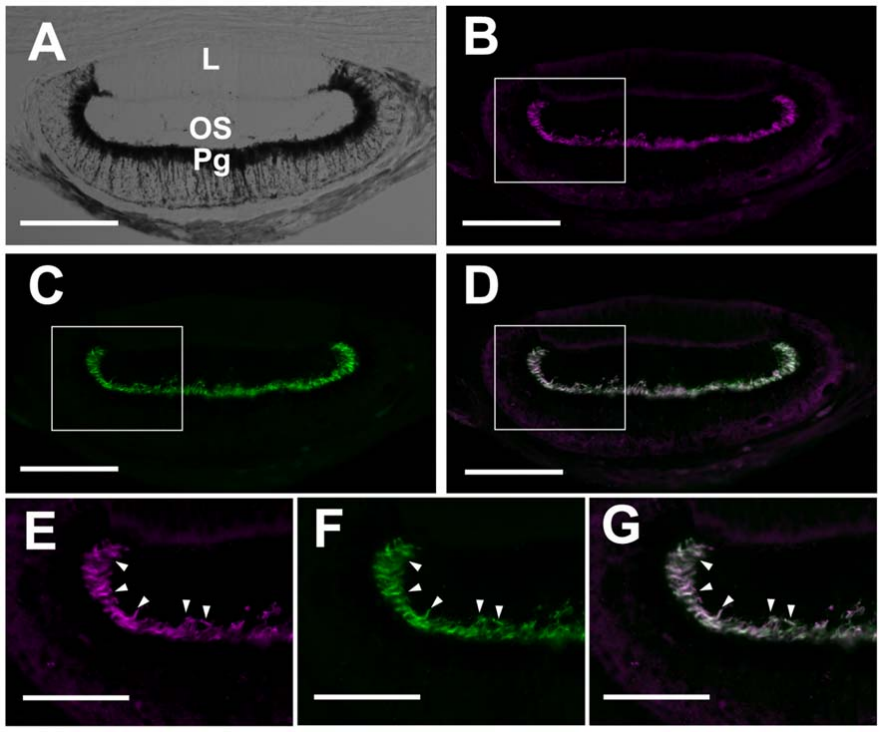
Colocalization of parapinopsin and parietopsin in the photoreceptor cells of the iguana parietal eye. (A) A Nomarski image of the parietal eye of the iguana. L, lens; OS, outer segments of the photoreceptor cells; Pg, pigment granule-rich region. (B and C) Immunofluorescence labeling of the photoreceptor outer segments in the parietal eye with the anti-iguana parapinopsin antibody (B, magenta) and the anti-iguana parietopsin antibody (C, green). (D) A merged image showing the colocalization of iguana parapinopsin and parietopsin in the photoreceptor cells. E, F and G are high magnifications of the boxed regions in B, C and D, respectively. The arrowheads show the outer segments of photoreceptor cells in the parietal eye. The scale bars indicate 100 µm in A, B, C and D and 50 µm in E, F and G.

### Generation of Anti-iguana Parapinopsin and Anti-iguana Parietopsin Antibodies

The antigens for the C-terminal regions of parapinopsin (49 amino acids) and parietopsin (48 amino acids), each of which show low amino acid identity to each other and those of other opsins (Figure S2), were prepared using the pMAL protein fusion and purification system (New England Biolabs) according to the manufacturer’s protocol. The anti-iguana parapinopsin antibodies were raised using mice and rabbits and anti-iguana parietopsin antibody was raised using mice, as the immunized animals.

### Examination of Antibody Immunoreactivity using Cultured Cells

For staining cells, the transfected cells were fixed in 100% Methanol for 5 minutes, treated with phosphate buffered saline (PBS) containing 2% bovine serum albumin (BSA) and 0.1% Tween-20 for 30 min at room temperature, incubated with primary rabbit or mouse antisera against iguana parapinopsin and parietopsin, respectively (diluted 1∶500) overnight at 4°C. Subsequently, the cells were incubated with Alexa Fluor 488 anti-rabbit or anti-mouse IgG (Molecular Probes, Eugene, OR, USA). For immunoblot analysis, the transfected cells were solubilized in sodium dodecyl sulfate (SDS) sample buffer (8% SDS, 20% Glycerol, 5% 2-Melcaptoethanol, 125 mM Tris/HCl pH 6.8). Aliquots (∼1 µg each) of the sample were applied to SDS-Poly-Acrylamide Gel Electrophoresis and separated proteins were transferred onto nitrocellulose membranes. The membranes were treated with PBS containing 2% BSA and 0.1% Tween-20, incubated with primary antibodies, rho 1D4 (hybridoma culture fluid), the antisera against iguana parapinopsin or parietopsin (diluted 1∶1000) overnight at room temperature. The detection was conducted with VECTASTAIN ABC Kit (Vector Laboratories).

### Immunohistochemistry

The iguanas were quickly decapitated, and their parietal eyes, pineal organs and eyes were isolated with a small piece of adjacent tissue. They were fixed in 4% paraformaldehyde in 100 mM sodium phosphate buffer (PB, pH 7.4) overnight at 4°C, cryoprotected by immersion in 100 mM PB containing increasing concentrations of sucrose (15 and 30%), embedded in OCT compound (Sakura, Tokyo, Japan) and sectioned at 30 µm. Primary antisera were diluted in PBS (pH 7.4) containing 0.1% Tween-20 (PBS-T), 0.5% Triton X-100 and 10% normal goat serum and secondary antibodies were diluted in PBS-T. The sections were incubated overnight at 4°C with the primary rabbit antiserum against iguana parapinopsin (diluted 1∶500) and, the mouse antiserum against iguana parietopsin (diluted 1∶500). The mouse monoclonal antibody against transducin and gustducin (TF15, CytoSignal) was also used (diluted 1∶500). The sections were subsequently incubated with Alexa Fluor 488 anti-rabbit IgG (diluted 1∶500) or Alexa Fluor 594 anti-mouse IgG (diluted 1∶500) for 5·h at room temperature. In the case of double staining using the primary rat antiserum against side-blotched lizard parietopsin [13] and the primary mouse antiserum against iguana parapinopsin, the sections were incubated overnight at 4°C with the primary rat antiserum against side-blotched lizard parietopsin (diluted 1∶200) and subsequently incubated with Cy3-conjugated anti-rat IgG (diluted 1∶500; Jackson Immunoreseach) for 5 h at room temperature. After wash with PBS-T, the sections were incubated overnight at 4°C with the primary mouse antiserum against iguana parapinopsin (diluted 1∶500). The sections were then incubated with Alexa Fluor 488 anti-mouse IgG (highly cross-adsorbed) (diluted 1∶500) for 5 h at room temperature.

## Results

We searched for the parapinopsin gene in the genome database for the green anole to investigate whether reptiles have parapinopsin, and found gene fragments coding for an opsin similar to parapinopsin. Phylogenetic analysis including vertebrate visual and non-visual opsins showed that the candidate anole gene clustered with the lamprey and frog parapinopsins (Figure 1), indicating that it is anole parapinopsin. With degenerate primers based on the deduced amino-acid sequence of anole parapinopsin, we successfully obtained the full-length parapinopsin cDNA of iguana (Figure S1). The iguana parapinopsin amino-acid sequence has ∼60% identity to that of the lamprey parapinopsin, with both clustered in the phylogenetic tree with high bootstrap support (99%) (Figure 1). Thus, parapinopsin is present in reptiles as in lampreys, fishes and frogs.

By RT-PCR analyses on iguana brain, pineal organ, parietal eye and lateral eye cDNAs, we found that parapinopsin is specifically detected in the parietal eye, in contrast to the UV cone pigment, which is detected only in the eye (Figure 2). Interestingly, iguana parapinopsin is not detected in the pineal organ, unlike the situation in lamprey and catfish [11], [12]. We also looked for pinopsin and parietopsin, blue- and green-sensitive pigments, respectively, previously found in the parietal eye of the side-blotched lizard [13]. RT-PCR suggested the presence of parietopsin, but interestingly not pinopsin, in the iguana parietal eye (although the latter was present in the pineal) (Figure 2).

Next, we investigated the spectroscopic characteristics of iguana parapinopsin and parietopsin. Iguana parapinopsin was successfully expressed in cultured cells and reconstituted as a functional pigment. The difference spectrum (before minus after UV light irradiation) of the pre-purified parapinopsin shows that the dark state of iguana parapinopsin, which has an absorption maximum in the UV region, is converted by UV light to a photoproduct with an absorption maximum in the visible light region (Figure 3A). This spectral change is similar to that for UV-sensitive lamprey parapinopsin (Figure 3A inset) [19], suggesting that iguana parapinopsin is also a UV-sensitive pigment, although we could not determine the absorption maximum of iguana parapinopsin due to the low expression level of the protein in cultured cells. We also investigated iguana parietopsin and found that this pigment has an absorption maximum at 522 nm (Figure 3B), indicating that it is a green-sensitive pigment and the absorption maximum is almost identical to the parietopsin of the side-blotched lizard [13].

To localize parapinopsin and parietopsin in the iguana parietal eye, we raised antibodies against C-terminal regions of iguana parapinopsin (49 amino acids) and parietopsin (48 amino acids) (Figure S2), because their C-terminal sequences are quite different from each other and from those of other opsins. The anti-parapinopsin and anti-parietopsin antibodies specifically labeled cultured cells expressing iguana parapinopsin and parietopsin, respectively (Figure 4A-D). They also labeled specifically ∼38 k and ∼39 k peptides in SDS-extract from transfected cells and their apparent molecular masses are similar to ones calculated based on amino acid sequences of parapinopsin and parietopsin, respectively (Figure 4E). In addition, no immunoreactivities of the antibodies were detected in the iguana pineal organ where pinopsin is expressed (Figure 2) and the retina having several kinds of visual pigments in rods and cones (Figure S3). It can be also speculated that encephalopsin (OPN3), melanopsin (OPN4), and neuropsin (OPN5) homologues exist in non-visual retinal cells according to observations for other lower vertebrates [20]–[23]. These results strongly suggested that these antibodies specifically recognized parapinopsin and parietopsin, respectively, and allowed us to investigate localization of parapinopsin and parietopsin in the parietal eye with these antibodies. The anti-parapinopsin and anti-parietopsin antibodies labeled the distal part of the photoreceptor layer of the iguana parietal eye retina, indicating that both parapinopsin and parietopsin are localized in the outer segments of the photoreceptor cells (Figure 5A-C, E, F). Remarkably, double-stained images show the colocalization of parapinopsin and parietopsin in the same photoreceptor outer segment (Figure 5D and G), suggesting that parapinopsin and parietopsin function in the same photoreceptor cells in the iguana parietal eye. The colocalization was also confirmed by confocal images, stained with the antibodies against iguana parapinopsin and side-blotched lizard parietopsin [13] (Figure S4).

## Discussion

In this paper, we reported the presence of parapinopsin in the reptile lineage (Figure 1). The spectroscopic analysis indicates that iguana parapinopsin is also a UV-sensitive pigment, similar to lamprey parapinopsin (Figure 3A). We also showed that iguana parapinopsin is localized in the outer segments of the parietal eye photoreceptors (Figures 5 and S4). Because the outer segment is a specialized region for light-sensing, we conclude that parapinopsin underlies the UV reception of the iguana parietal eye. An electrophysiological study revealed that the parietal eye of the green iguana exhibited antagonistic chromatic responses between UV and visible light, including blue and green light [7], suggesting that parapinopsin is the UV pigment for wavelength discrimination in the parietal eye. Interestingly, in the iguana, parapinopsin is not detected in the pineal organ (Figure 2), in contrast to the expression of parapinopsin in the pineal and parapineal organs in lamprey and catfish [11], [12]. It was proposed that the parietal eye, found only in lizards, is homologous to the parapineal organ of other lower vertebrates [14], [15]. Accordingly, the current findings suggest that the expression of parapinopsin in the pineal organ has been lost in the lizard lineage, whereas the expression in the parapineal organ has been retained, despite the drastic morphological change of this organ to the parietal eye during evolution. The loss of the UV-sensitive pigment in the pineal organ of the iguana suggests that the wavelength discrimination involving UV reception is achieved only in the parietal eye. During the evolution of the parietal eye, which is the most complex and highly-developed photoreceptor organ among the pineal-related organs, functional specialization for wavelength discrimination might have occurred in the pineal-related organs.

We also found that iguana parietopsin has an absorption maximum at 522 nm (Figure 3B) and is colocalized with parapinopsin in the same photoreceptor outer segments in the parietal eye (Figures 5 and S4), suggesting that parietopsin underlies the green light reception for wavelength discrimination in the iguana parietal eye, as in the case of the side-blotched lizard parietal eyes [13]. In the side-blotched lizard, the blue-sensitive photopigment pinopsin and parietopsin are colocalized in the outer segments of a single photoreceptor cell and act antagonistically to detect the ratio of blue to green light. However, in the iguana parietal eye, RT-PCR analyses suggested that the expression level of pinopsin is much lower than those of parapinopsin and parietopsin. These observations revealed that the repertoire of pigments in the parietal eye could vary depending on lizard species. Interestingly, pinopsin is coupled to gustducin in the parietal eyes of the side-blotched lizard [13], and we recently found that parapinopsin is colocalized with transducin in the lamprey pineal organ [24]. Because gustducin and transducin are very similar [25] and parapinopsin activates the Gi type of G protein [19], [26], which is similar to gustducin and transducin, pinopsin and parapinopsin might trigger a similar G protein-mediated signal transduction cascade. Therefore, parapinopsin (instead of pinopsin) and parietopsin may act antagonistically to detect the ratio of UV to visible light in a single photoreceptor cell in the iguana parietal eye, with a mechanism similar to that employed by pinopsin and parietopsin in the side-blotched lizard [13].

In this paper, we found that parapinopsin serves as a UV sensitive photopigment for the wavelength discrimination in the lizard parietal eye, like the pineal organs of lamprey. This finding supports the idea that parapinopsin is a common photopigment underlying UV-sensitivity of the wavelength discrimination in the pineal-related organs of lower vertebrates.

## Supporting Information

Figure S1 **Alignment of amino acid sequences of iguana opsins and other vertebrate opsins.** Seven putative membrane spanning domains (TM I-VII) deduced from a comparison with bovine rhodopsin (K00502) were shown by horizontal bars. Amino acid residues that are highly conserved in opsins are highlighted [27]. The lysine residues which bind to the retinal chromophore are shown in white letters on black. Two cysteine residues, which are typical for G-protein coupled receptors, are boxed. Asterisks indicate the candidates for counterion residues.
(TIF)

Figure S2 **Comparison of amino acid sequences of C-terminal regions of reptilian opsins.** C-terminal regions of iguana parapinopsin and parietopsin, which were used for antibody generation (horizontal bar), show low similarity to each other and those of other reptilian opsins. ig_pp, iguana parapainopsin; ig_prt, iguana parietopsin; ig_pin, iguana pinopsin; an_lw, anole long wavelength-sensitive opsin (U08131); an_sw1, anole short wavelength-sensitive opsin1 (AF134194); an_sw2, anole short wavelength-sensitive opsin2 (AF133907); an_mw, anole middle wavelength-sensitive opsin (S79167); an_rh, anole rhodopsin (L31503).
(TIF)

Figure S3 **Specific Immunoreactivity of antibodies to parapinopsin and parietopsin in the parietal eye.** Immunoreactivity of anti-transducin/gustducin (TF15) (A-C), anti-parapinopsin (D-F) and anti-parietopsin (G-I) antibodies in the iguana retina (A, D, G), pineal organ (B, E, H) and parietal eye (C, F, I). OS indicates the outer segments of photoreceptor cells in the retina and parietal eye. The scale bars indicate 100 µm.
(TIF)

Figure S4 **Confocal images showing colocalization of parapinopsin and parietopsin.** (A and B) Immunofluorescence labeling of the photoreceptor outer segments in the iguana parietal eye with the antibodies to the iguana parapinopsin (A, green) and side-blotched lizard parietopsin (B, magenta). (C) A merged image showing the colocalization of parapinopsin and parietopsin in the same photoreceptor outer segments in the iguana parietal eye. The scale bars indicate 25 µm.
(TIF)
