# Supplementary figures and images for: Expression of UV-Sensitive Parapinopsin in the Iguana Parietal Eyes and Its Implication in UV-Sensitivity in Vertebrate Pineal-Related Organs
Source: PLoS One. 2012 Jun 14;7(6):e39003. doi: 10.1371/journal.pone.0039003 (PMC3375259; doi:10.1371/journal.pone.0039003)

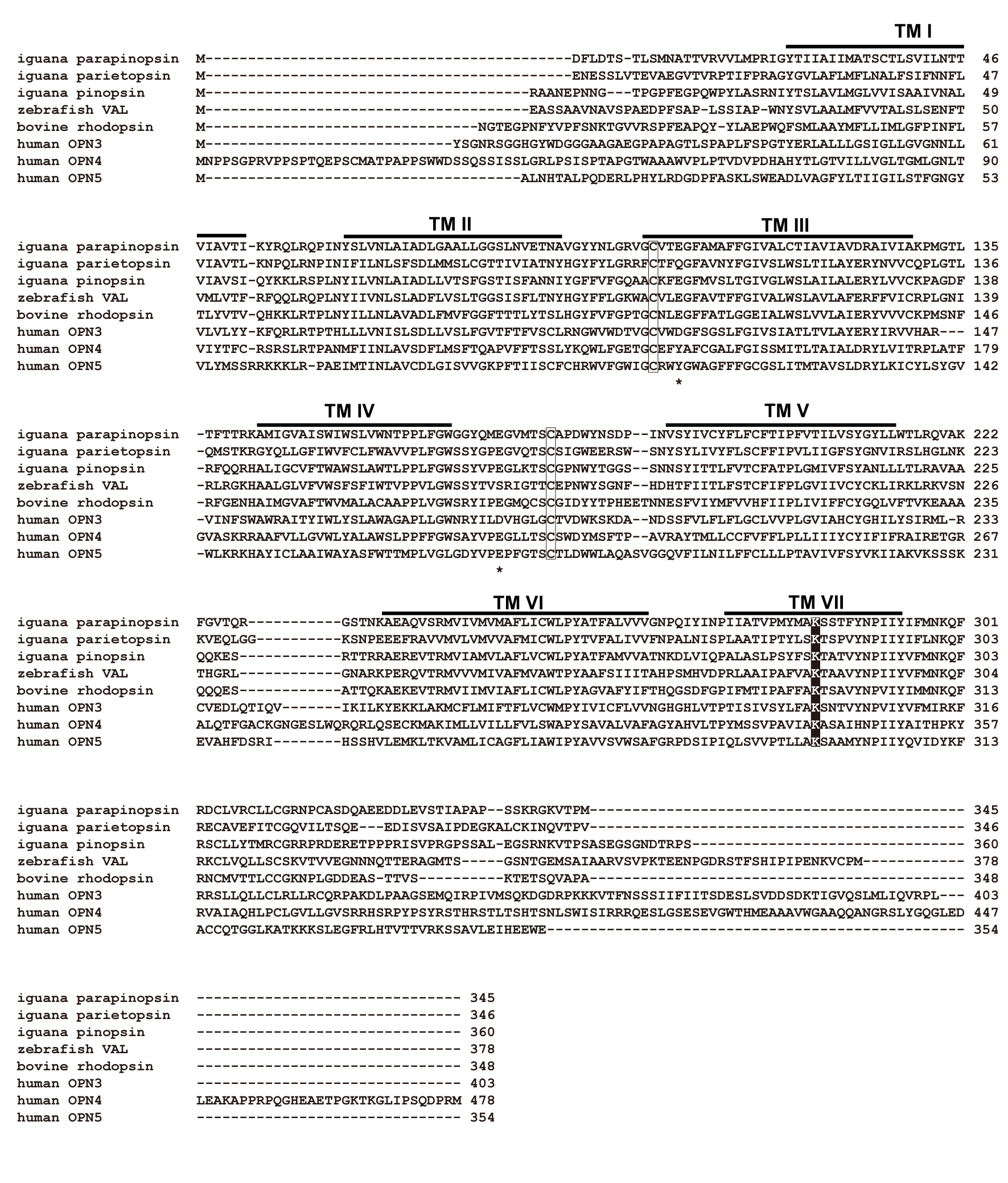

Supplement: Figure S1 — Alignment of amino acid sequences of iguana opsins and other vertebrate opsins. Seven putative membrane spanning domains (TM I-VII) deduced from a comparison with bovine rhodopsin (K00502) were shown by horizontal bars. Amino acid residues that are highly conserved in opsins are highlighted [27]. The lysine residues which bind to the retinal chromophore are shown in white letters on black. Two cysteine residues, which are typical for G-protein coupled receptors, are boxed. Asterisks indicate the candidates for counterion residues. (TIF) [file pone.0039003.s001.tif]

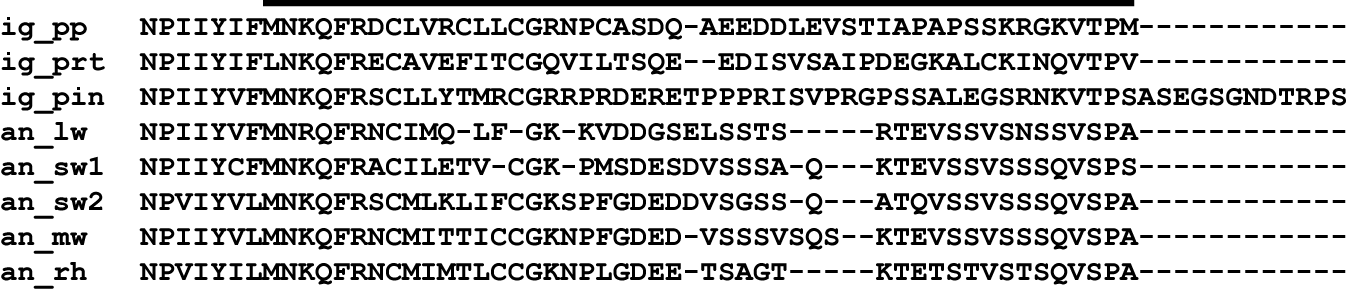

Supplement: Figure S2 — Comparison of amino acid sequences of C-terminal regions of reptilian opsins. C-terminal regions of iguana parapinopsin and parietopsin, which were used for antibody generation (horizontal bar), show low similarity to each other and those of other reptilian opsins. ig_pp, iguana parapainopsin; ig_prt, iguana parietopsin; ig_pin, iguana pinopsin; an_lw, anole long wavelength-sensitive opsin (U08131); an_sw1, anole short wavelength-sensitive opsin1 (AF134194); an_sw2, anole short wavelength-sensitive opsin2 (AF133907); an_mw, anole middle wavelength-sensitive opsin (S79167); an_rh, anole rhodopsin (L31503). (TIF) [file pone.0039003.s002.tif]

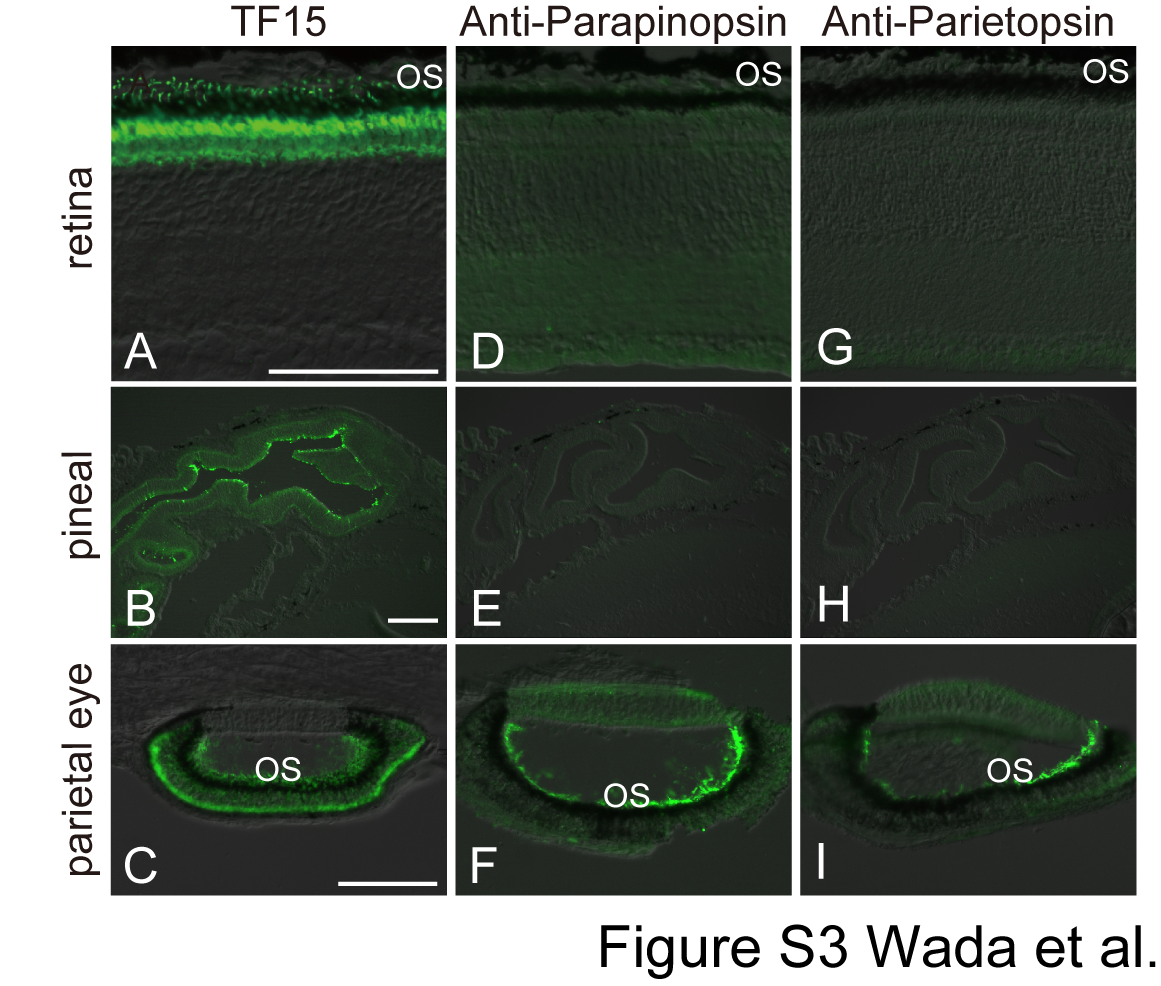

Supplement: Figure S3 — Specific Immunoreactivity of antibodies to parapinopsin and parietopsin in the parietal eye. Immunoreactivity of anti-transducin/gustducin (TF15) (A-C), anti-parapinopsin (D-F) and anti-parietopsin (G-I) antibodies in the iguana retina (A, D, G), pineal organ (B, E, H) and parietal eye (C, F, I). OS indicates the outer segments of photoreceptor cells in the retina and parietal eye. The scale bars indicate 100 µm. (TIF) [file pone.0039003.s003.tif]

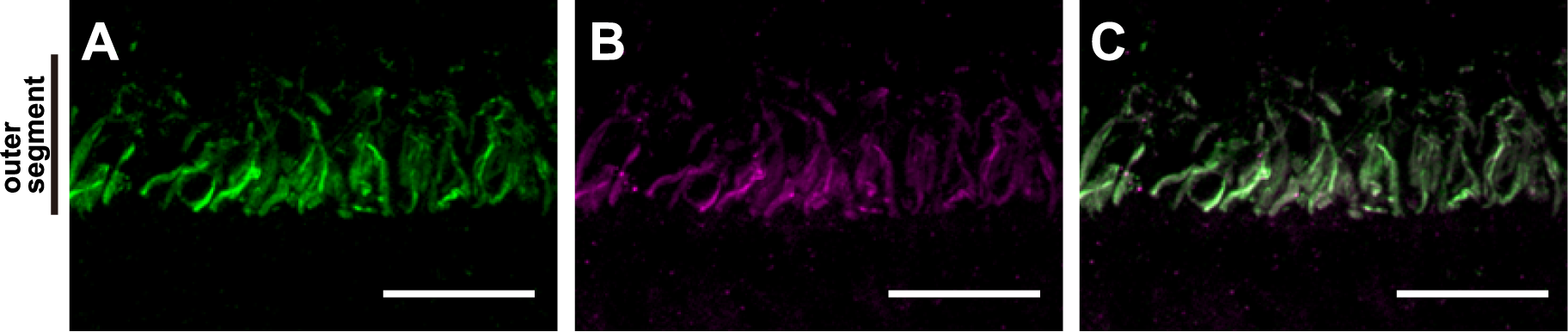

Supplement: Figure S4 — Confocal images showing colocalization of parapinopsin and parietopsin. (A and B) Immunofluorescence labeling of the photoreceptor outer segments in the iguana parietal eye with the antibodies to the iguana parapinopsin (A, green) and side-blotched lizard parietopsin (B, magenta). (C) A merged image showing the colocalization of parapinopsin and parietopsin in the same photoreceptor outer segments in the iguana parietal eye. The scale bars indicate 25 µm. (TIF) [file pone.0039003.s004.tif]
